# Sustainable behavior begins with sustainable attitudes: Data to assess the relationships between demographics, concerns about transport-related pollution, and willingness for action

**DOI:** 10.1016/j.dib.2024.110446

**Published:** 2024-04-18

**Authors:** Francisco Alonso, Cristina Esteban, Mireia Faus, Sergio A. Useche

**Affiliations:** a INTRAS (Research Institute on Traffic and Road Safety), University of Valencia, Spain; b Faculty of Psychology, University of Valencia, Valencia, Spain

**Keywords:** Mobility, Attitudes, Environmental concern, Transport-related pollution, Willingness for action

## Abstract

This Data in Brief (DiB) article addresses the relationships among individuals’ transport habits, perceptions, and attitudes regarding transport-related pollution, as well as their willingness to act for environmental change. There are presented descriptive statistics, basic comparisons, and bivariate correlations among the core variables of the study. Additionally, the attached dataset appends information from a nationwide sample of 1,250 citizens in the Dominican Republic, with sex, age and habitat distribution proportional to the national census. The research database contains the full set of questions and responses to the research questionnaire, which is also attached to the supplementary files along with its codebook, making it possible to conduct further data explorations for research-related, divulgation, and educational purposes. For more information about the root study, it is advisable to read the paper “Who wants to change their transport habits to help reduce air pollution? A nationwide study in the Caribbean”, published in *Journal of Transport & Health* [1].

Specifications Table

**Table:** 

Subject	Psychology
Specific subject area	Urban mobility, users’ behavior, and perceptions about pollution
Data format	Filtered and Analyzed
Type of data	Tables with the data of the differences of own concern and willingness for action according to type of user. Graphs representing a Pearson correlation analysis of the study variables. Database
Data collection	Participants responded to a nationwide survey applied in a face-to-face approach, using computer-assisted interviews at public locations.
Data source location	Data were collected in the Dominican Republic (Central America)
Data accessibility	The presented data is derived from the original database reported in the article. It also contains the full database obtained for the study. It contains the .CSV format dataset, the root questionnaire, and the study codebook. Data are fully accessible at the permanent link (Harvard Dataverse): https://doi.org/10.7910/DVN/JWZVZ7
Related research article	Alonso, F., Faus, M., Esteban, C., & Useche, S. A. (2023). Who wants to change their transport habits to help reduce air pollution? A nationwide study in the Caribbean. Journal of Transport & Health, 33, 101,703. https://doi.org/10.1016/j.jth.2023.101703[1]

## 1 Value of the Data

• These data provide information about the relation between pollution awareness, willingness for action, and transport habits of Dominican Republic citizens.
• The data are eligible for comparison with those obtained in other countries, especially in Latin America, with which they share socio-demographic features similar to the Dominican Republic.
• The data can be used by both research bodies and responsible entities of the country for the design of specific mobility and transport plans.
• The data could be useful to design awareness campaigns and promote massive, active, and sustainable means of transport.

## 2 Background

Give that transport-related air pollution remains a pressing environmental concern, the United Nations’ 2030 Agenda for Sustainable Development has prioritized energizing urban planners, policymakers, and several other stakeholders to address it as part of the current sustainable mobility goals [2]. However, and although it may be thought that this is limited to technical measures, such as improving energy efficiency, restricting car travel to privilege cycling or public transportation, or manufacturing cleaner or more environmentally-friendly vehicles, recent evidence suggests that the greatest challenge may lie in the attitudinal sphere, that is, engaging the population to make individual “sacrifices” that may favor the fight against climate change and environmental pollution in general [3,4].

Furthermore, as an additional constraint for Low- and Middle-Income Countries (LMICs), environmentally-related attitudinal factors and their subsequent behavioral outcomes remain scarcely investigated compared to High-Income economies, whose specific strengths, shortcomings, and present challenges may substantially differ. Indeed, to the best of our knowledge, this constitutes the first study addressing this matter in the Caribbean region. For these reasons, this data article aims to complemente the insights added by us in Alonso et al. (2023) [1], that evaluates assess the effect of individual factors, transport habits, and pollution-related concerns on the individual's will to act to reduce air pollution in the Dominican Republic.

The findings of this research, published in *Journal of Transport & Health*, highlight that both personal and societal concerns regarding pollution have an impact on individuals’ self-reported likelihood to take action against air pollution through changes in their transportation habits, albeit in varying manners. Remarkably, individuals’ personal environmental concerns increase their self-reported likelihood to take action (e.g., shifting to public or active transport, reducing trips). However, merely acknowledging a broader societal environmental concern without a significant degree of individual worry seems to have a ‘side effect’, namely, diminishing individuals' willingness to engage in pro-environmental behavioral changes. Accordingly, the current data article presents a set of additional analyses addressing the links among these study variables, as well as full access to the study data base, as described in the following sections.

## 3 Data Description

The dataset file appended in this data article, made open by the study authors, provides other researchers the possibility to analyse the relationships among our study variables, including daily transport habits, perceptions regarding the pollution generated by traffic and vehicle mobility, and self-reported willingness to “sustainabilize” transport habits among citizens residing in the Dominican Republic. In addition, presented below are some descriptive analyses comparing the study outcomes among different groups of individuals cathegorized by age and licensing status.

**Fig. 1:**
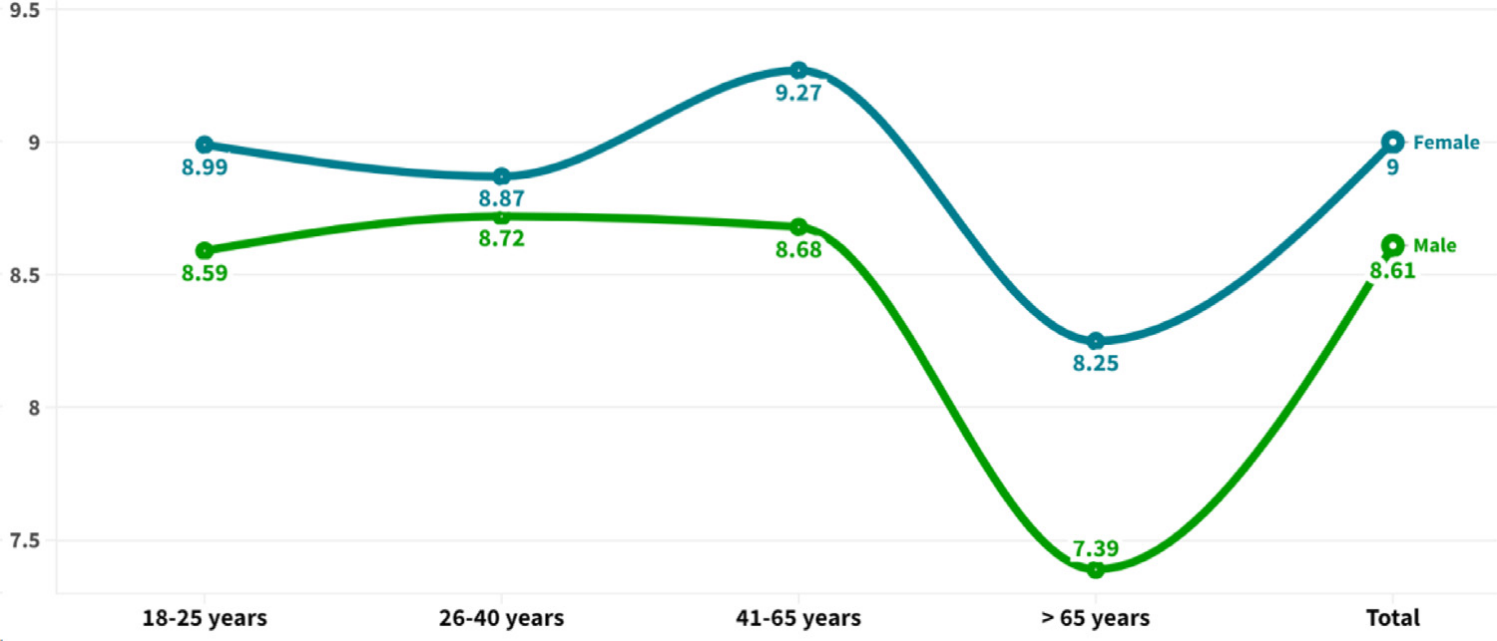
Differences in the degree of awareness about air pollution based on gender and age.

**Fig. 2:**
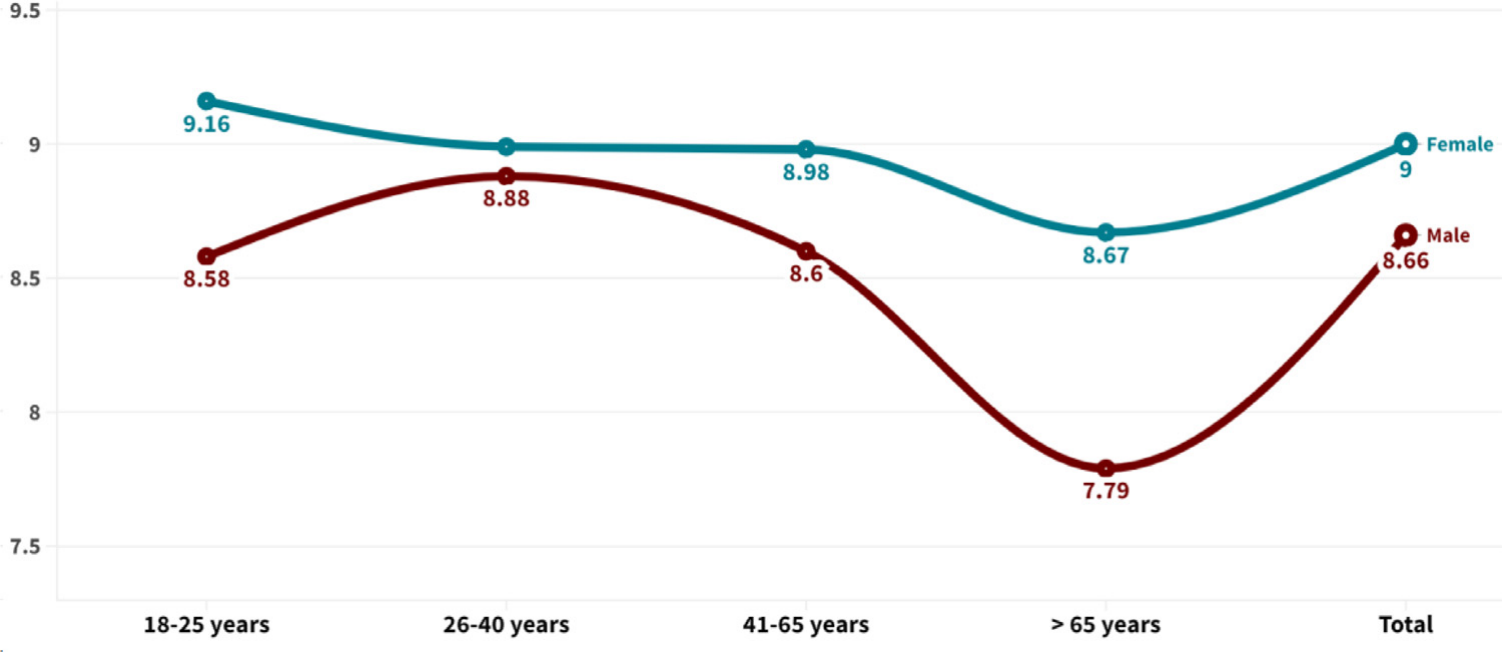
Differences in willingness for action to reduce pollution based on gender and age.

**Fig. 3:**
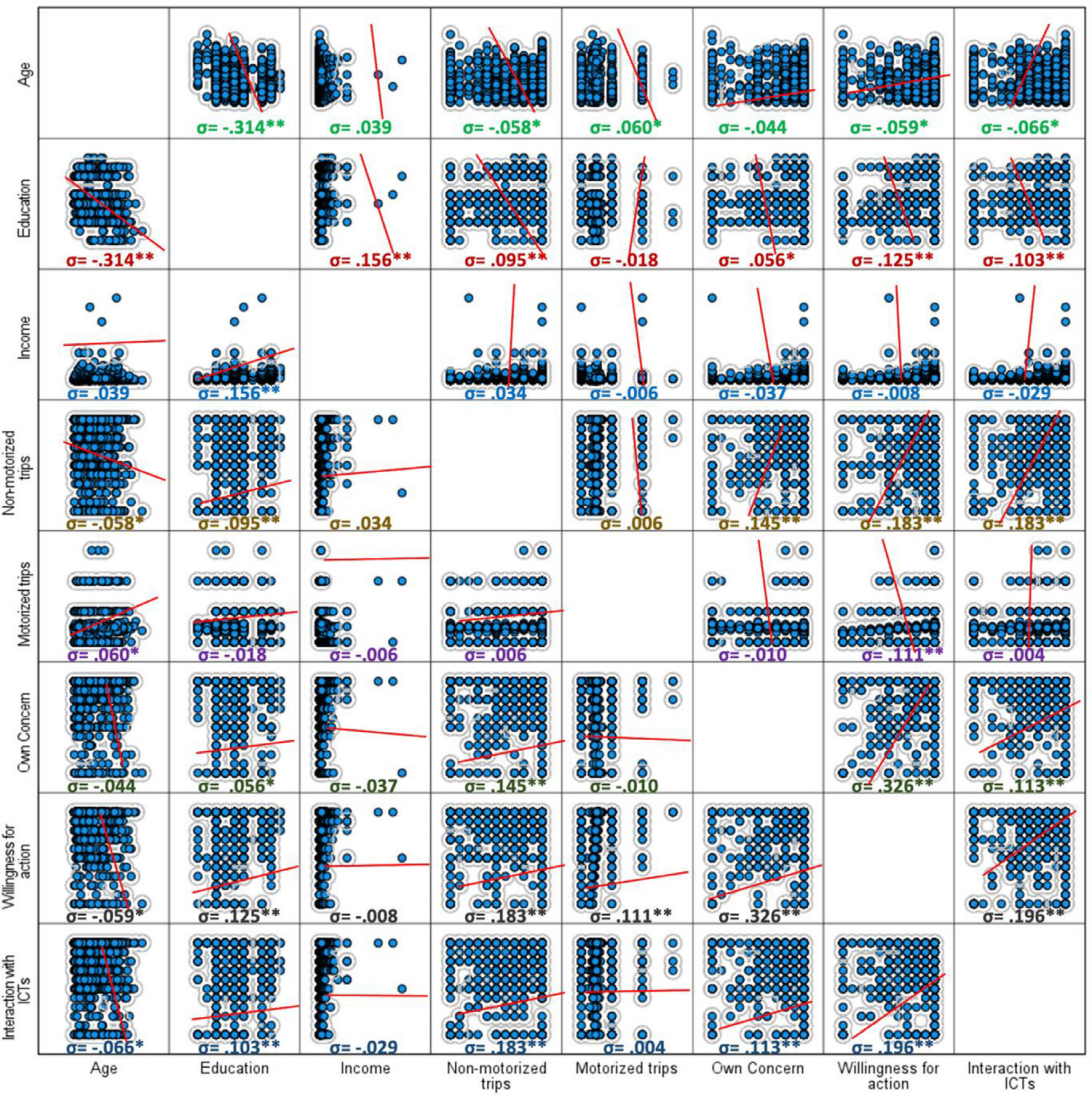
Graphical bivariate correlations between factors included in the dataset.

Fig. 1, Fig. 2 graphically display the differences based on age group and gender of the variables “own concern” and “willingness for action”. Table 1 shows the differences in the degree of concern about pollution according to the most common mobility user types in the country (i.e., private drivers and public transport users). Table 2 presents the differences among these groups of users concerning their self-reported degree of willingness to act to reduce pollution levels. In terms of bivariate associations, Fig. 3 shows a Pearson correlation matrix among the study variables, including those related with individuals’ own concern, willingness for action, interaction with ICTs, frequency of motorized and non-motorized trips, and basic socio-demographic variables, i.e., age, education, and individual income collected in the study.

**Table 1:** Degree of concern about air pollution among Dominican citizens.

Factor			N	M	SD
Degree of concern about air pollution (full Dominican citizens sample).			1250	8.80	2.45
Sociodemographic variables					

Holds a driving license?	t_(1248)_ = 0.383 *p* = .351	Yes	270	8.86	2.134
		No	980	8.79	2.541
Regular driver?⁎	t_(1248)_ = −0.915; *p* = .180	Yes	464	8.72	2.388
		No	786	8.85	2.498
Habitual public transport user?⁎	t_(1248)_ = 1.972; *p* = .024	Yes	734	8.92	2.336
		No	516	8.64	2.615

*Notes*:

⁎ Regular/habitual user was defined as those using the transport means a minimum of 3 times a week.

**Table 2:** Self-reported degree of willingness for action to reduce transport-related pollution.

Factor			N	M	SD
Degree of willingness for action to reduce vehicle-generated pollution			1250	8.83	2.33
Sociodemographic variables					

Holds a driving license?	t_(1248)_ = −0.32; *p* = .487	Yes	270	8.83	2.093
		No	980	8.83	2.394
Regular driver?⁎	t_(1248)_ = −1.577; *p* = .057	Yes	464	8.70	2.446
		No	786	8.91	2.260
Habitual public transport user?⁎	t_(1248)_ = 1.591; *p* = .057 .	Yes	734	8.92	2.244
		No	516	8.71	2.448

*Notes*:

⁎ Regular/habitual user was defined as those using the transport means a minimum of 3 times a week.

## 4 Experimental Design, Materials and Methods

### 4.1 Participants

In this cross-sectional research, the full study sample was composed of a stratified sample of 1250 inhabitants of the Dominican Republic. The sampling distribution was proportional to the national census of the country (provided by the National Statistics Office) in terms of gender, age (adults; above the age of 18) habitat and province (all provinces of the country were covered). The average age of participants was *M* = 39.4 years (*SD*= 15.4) [18–92 range], and the gender proportion was 50 % (*n* = 625) males and 50 % (*n* = 625) females.

As for the nationwide representativeness of the data, it is worth mentioning that the study sample was based on a minimum size estimated at 680 individuals, calculated under the following statistical parameters and assumptions: a confidence level of 95 %, a maximum margin of error of 5 % (α = 0.05), and a beta (β) of 0.20, allowing for a statistical power of 80 %. In terms of demographic features, the sample was balanced in terms of participants’ sex and distributed fairly in terms of age and public transport usage, as well as driving status (i.e., holding or not a driving license), all proportionally distributed based on population data.

### 4.2 Questionnaire structure and variable definition

The questionnaire used for this study addressed issues related to daily mobility habits, the use of public and private transport, air pollution, road safety, and possible actions and prevention measures. The variables contained in the dataset are defined as follows:

*Age/age range:* The number of completed years of age the participant had at the time of responding to the survey. While “age” appends the continuous values, “agen range” splits it in four groups: early adulthood (18–24 years), early middle age (26–40 years), late middle age (41–65 years), and late adulthood (>65 years).

*Gender:* The gender label (Male/Female/Other)ith which participants identify. It is important to clarify that no participant identified as "other” o “non-binary". In addition to aligning with the typology of the reference population census (which also does not include third genders), in the Dominican Republic, there is a fairly conservative culture regarding these matters, so the result corresponds with what would be expected.

*Habitat:* Type of area (Urban/Rural) where the participant's primary residence is located. In the case of the country, regions are classified for census purposes as “urban “or “rural” based on population density. Therefore, these two values were used accordingly.

*Education:* Self-reported level of educational attainment (completed) by participants at the time of the survey. It has a range of 10 values ranging from “No formal studies but able to read and write” (minimum needed to participate) to “University postgraduate or doctorate”.

*Income:* Continuous variable representing the number of units of the local currency (Dominican Pesos; approximately 1:0.017 USD) received by the participant on a monthly basis.

*License:* Corresponds to a categorical variable (Yes/No) documenting partticipants’ licensing status, i.e., holding (or not) valid driving license, regardless of its tyopology, at the time of responding to the survey.

*Driver:* Categorizes whether the person, if being a driver of any type of vehicle, drives a minimum of three times per week.

*Public Transport:* Categorizes whether the person, if being a public transport user, utilizes any of its modes a minimum of three times per week.

*Motorized trips:* Ordinal variable assessing the frequency with which the participant engages in motor vehicle trips each week, uning a four-point scale ranging from “almost never” to “sometimes a week”.

*Concern levels:* These indicators were assessed using scale ranging from 0 to 10, where 0 represents “no concern at all”, and 10 denotes “very high concern”. Two levels of analysis were established: *Own concern degree:* This factor was based on individuals’ self-reported awareness of air pollution caused by transportation, i.e., to what extent do they consider that transport contributes to air pollution. *Perceived social concern:* This indicator assesses individuals' perceptions of the level of concern regarding vehicle-related air pollution in their community, i.e., to what extent do they consider that their community member consider that transport contributes to air pollution.

*Willingness for action:* This variable refers to the degree to which participants are open to playing a part in decreasing atmospheric emissions through their personal behaviors. This aspect was assessed using a single item (i.e., To what extent would you be willing to modify your transportation habits to lessen air pollution, even if it involves significant changes in your daily mobility?), rated on a scale from 0 (not important at all) to 10 (very important).

*Interaction with ICTs:* The general extent of participants' interaction with Information and Communication Technologies (ICTs), such as smartphones, tablets, navigators, wearables, and other internet-connected devices, was measured in a 10-point single item ranging between “very scarce interaction” and “very high interaction”.

The data collection was carried out in collaboration with the Dominican government alongside the National Mobility Survey of the Dominican Republic, which is annually launched throughout the country by public agencies [5].

### 4.3 Data analysis

In the development of the present investigation based on populational self-reports, descriptive analyses were carried out to provide a thorough perspective of the degree of importance that the Dominican population gives to the transport-related pollution issue in their country, as well as the perceived impact of actions that would potentially reduce its impact in the region. Furthermore, Pearson bivariate correlation coefficients were used to identify possible statistical relations among the key indicators of the study, i.e., pollution perceptions, self-reported willingness for action, interaction with connected technologies (ICTs), basic transport habits, and sociodemographic variables. As for demographic comparisons, Student *t*-tests were used to assess potential differences in pollution-related appraisals based on individual transportation habits. Statistical analyses were carried out through the software © IBM SPSS (Statistical Package for Social Sciences) version 28.0.

## Limitations

Although this study had a wide coverage and used a stratified sample, some key fundamental limitations must be acknowledged. Firstly, our results are based on self-report questionnaire data [6]. In this study setting, biased or inaccurate answers can be provided by participants, especially considering that some potentially sensitive topics attaining enviuronmental issues (e.g., behavior and personal attitudes regarding pollution) are addressed in the study [7]. In addition, the present study does not conduct inferential analyses, which implies the impossibility of controlling for key variables such as income, habitat, educational attainment, and environmental confounders for assessing demographic-attitudinal relationships [8,9]. Finally, the number of variables in the study is limited, and other unaddressed factors that could potentially influence the participants’ perceptions could further help to improve the addressing of these issues.

## Ethics Statement

The involvement of the study partakers was voluntary and anonymous. After the explanation of the research objectives and considerations related to the research, all participants gave their informed consent before participating in the study (a copy of the informed consent is available alongside this paper). The study was approved by the Ethics Committee of the Research Institute on Traffic and Road Safety at the University of Valencia (IRB approval number: HE0002241120).

## CRediT authorship contribution statement

**Francisco Alonso:** Conceptualization, Supervision, Investigation. **Cristina Esteban:** Conceptualization, Investigation. **Mireia Faus:** Data curation, Writing – original draft, Writing – review & editing. **Sergio A. Useche:** Conceptualization, Data curation, Writing – review & editing.

## Data Availability

Transport-related pollution and willingness for action in the Domincan Republic (Original data) (Dataverse).
